# Non-Invasive Blood Glucose Estimation System Based on a Neural Network with Dual-Wavelength Photoplethysmography and Bioelectrical Impedance Measuring

**DOI:** 10.3390/s22124452

**Published:** 2022-06-12

**Authors:** Chih-Ta Yen, Un-Hung Chen, Guo-Chang Wang, Zong-Xian Chen

**Affiliations:** 1 Department of Electrical Engineering, National Taiwan Ocean University, Keelung City 202301, Taiwan; 2 Department of Electrical Engineering, National Formosa University, Huwei Township 632, Taiwan; 10865120@gm.nfu.edu.tw (U.-H.C.); 10865122@gm.nfu.edu.tw (G.-C.W.); 10965125@gm.nfu.edu.tw (Z.-X.C.)

**Keywords:** blood glucose estimation, photoplethysmography (PPG), bioelectrical impedance, principal component analysis (PCA), back-propagation neural network (BPNN)

## Abstract

This study proposed a noninvasive blood glucose estimation system based on dual-wavelength photoplethysmography (PPG) and bioelectrical impedance measuring technology that can avoid the discomfort created by conventional invasive blood glucose measurement methods while accurately estimating blood glucose. The measured PPG signals are converted into mean, variance, skewness, kurtosis, standard deviation, and information entropy. The data obtained by bioelectrical impedance measuring consist of the real part, imaginary part, phase, and amplitude size of 11 types of frequencies, which are converted into features through principal component analyses. After combining the input of seven physiological features, the blood glucose value is finally obtained as the input of the back-propagation neural network (BPNN). To confirm the robustness of the system operation, this study collected data from 40 volunteers and established a database. From the experimental results, the system has a mean squared error of 40.736, a root mean squared error of 6.3824, a mean absolute error of 5.0896, a mean absolute relative difference of 4.4321%, and a coefficient of determination (*R* Squared, *R*^2^) of 0.997, all of which fall within the clinically accurate region A in the Clarke error grid analyses.

## 1. Introduction

Diabetes is a chronic disease that occurs when a patient’s pancreas is no longer capable of producing insulin or the patient’s body is unable to fully utilize the insulin it produces, leaving the body incapable of regulating blood glucose levels. Symptoms of diabetes include increased thirst, hunger, and urination frequency, and individuals with diabetes are at increased risk of several severe complications including retinopathy, kidney diseases, neuropathy, and cardiovascular diseases [1]. These complications can lead to reduced quality of life and long-term disability; therefore, preventive medicine is crucial for the early detection and treatment of diabetes. To facilitate early detection and treatment, regular blood glucose monitoring should be performed, enabling people to seek more accurate testing in hospitals in the early stages of diabetes.

Measurement of blood glucose generally requires patients to use a lancet and blood sugar test strip to collect blood from their fingers [2], after which an electronic device is used to measure and convert the data into a blood glucose value. However, frequent pricking of the fingers can be painful if regular monitoring is required, rendering the procedure unsuitable for repeated use. Finger pricking can also lead to infection, tissue damage, or reduced patient compliance [3]. Despite these shortcomings, invasive glucose monitoring is currently the most dominant means of measuring blood sugar due to a lack of noninvasive blood glucose meters that are as reliable and inexpensive as invasive ones. Various studies are currently being conducted using different noninvasive continuous blood glucose measurement methods that can be as accurate as invasive approaches at relatively low cost [4,5]. Currently, noninvasive blood glucose measurements are primarily conducted through four approaches: measurement of acoustic waves, microwaves, and electrical and optical signals. This study used both electrical bioelectrical impedance values and optical photoplethysmography (PPG) to obtain blood glucose-related parameters and predict blood glucose values.

PPG is a noninvasive physiological sensing technology that detects blood volume changes optically and can acquire signals from multiple body parts. The measurement sites affect PPG signals, and not all body parts are suitable for measuring PPG signals, which are best measured at the fingertips and earlobes [6]. Several studies have identified the relevance of noninvasive blood glucose measuring technologies using PPG signals for blood glucose analyses. Because blood glucose has different absorption rates for light sources of different wavelengths, Sen et al. proposed a novel design to detect blood glucose through three sensors using light sources with wavelengths of 940 nm, 660 nm, and 660 nm. They used two sensors (940 nm and 660 nm) to obtain the refraction signal and a third sensor (660 nm) to acquire the reflection signal, after which they conducted data analyses using analyses of variance [7]. Deepthi et al. used a 940 nm infrared light and proposed a blood glucose monitoring method that outperformed the invasive measurement approaches used by hospitals with a percentage error of ±2.5%, indicating that the model can favorably predict blood glucose concentrations [8].

Following the rise of artificial intelligence, some researchers have focused on using neural networks to evaluate PPG signal characteristics. Hamdi et al. used artificial neural networks to predict blood glucose levels. They utilized tanh as the activation function and achieved a mean square error of 6.43 mg/dL [9]. Prabhu et al. used classifiers such as deep belief networks, feedforward neural networks, decision trees, logistic regression, random forests, and support vector machines to compare predicted blood glucose values. They used the diabetes data set of UCI and determined that the deep belief networks had higher accuracy because they displayed the highest precision and recall rate [10]. Manurung et al. used transmitted infrared light with a wavelength of 940 nm that was preprocessed using high-pass and low-pass filters and used the maximum value, minimum value, sex, weight, height, skin color index, and finger width of the patient as inputs. Through neural network training, their model produced an absolute mean error of 5.855 mg/dL [11]. Hina et al. introduced a wearable blood glucose monitoring system that used infrared light with a single wavelength to collect PPG signals and incorporated machine learning regression to address the problem of facial motion artifacts. The authors compared the methods of Savitzky–Golay (SG) filtering, average filtering, and wavelet transform. Because all three filtering methods could effectively remove noise, they selected smooth filtering because it required less cost and computing time to complete signal preprocessing [12].

Bioelectrical impedance measuring technology involves using the electrical characteristics (impedance, phase, and dielectric constant) of biological tissues and organs and their changes to obtain information about the physiological and pathological status of the human body including body composition. The use of different bioelectrical impedance measuring technologies requires different excitation signal frequencies. In recent years, common bioelectrical impedance research methods include single-frequency bioelectrical impedance analyses, multifrequency bioelectrical impedance analyses, and bioelectrical impedance frequency sweep analyses. Zeng et al. measured the deionized aqueous solutions and saline solutions of glucose aqueous solutions in the frequency range of 500 kHz to 5 MHz at 25 °C using an impedance analyzer, and established an e-Cole model for each type of glucose. The resulting measurement coefficient of determination was 0.99, demonstrating the practical potential of low-frequency noninvasive blood glucose measurements [13]. Li et al. obtained the conductivity and dielectric constant of aqueous solutions with different glucose concentrations through an impedance analyzer in the frequency range of 1 kHz to 1 MHz. The results indicated that the dielectric constant did not differ significantly between aqueous solutions of different glucose concentrations, and the conductivity of the solutions varied with the increase in glucose concentration [14].

In addition, the combination of PPG signals and bioelectrical impedance has been verified to yield accurate results. Fouad et al. proposed a low-cost, high-precision noninvasive glucose monitoring system by combining multiwavelength infrared spectroscopy and bioelectrical impedance frequency sweep. In their method, bioelectrical impedance was primarily measured through frequency scanning from 10 to 100 kHz with an interval of 10 kHz, whereas infrared spectroscopy used three wavelengths (850 nm, 880 nm, and 940 nm). The correlation coefficient of their system was 0.91805, which fell in region A of the Clarke error grid analysis (EGA) [15]. Nanayakkara et al. measured bioelectrical impedance through 940 nm infrared light and a frequency of 3 to 100 kHz and compared the obtained features with least squares regression and neural network algorithms. The results revealed that the least squares regression was the superior algorithm and that the combination of infrared light and bioelectrical impedance yielded improved accuracy [16]. Pathirage et al. obtained a multiwavelength near-infrared light spectrum from multi-wavelength infrared light and extracted the features of bioelectrical impedance every 0.5 kHz from 50 to 100 kHz. Furthermore, they used a commercially available glucose meter to obtain the blood glucose of research participants, which was used to train a random forest regression model, obtaining an accuracy rate of 90.7% [17].

In the aforementioned studies, bioelectrical impedance was mostly analyzed in the form of an impedance spectrum, which can provide sufficient information and features and is thus conducive to the analyses of blood glucose through a back-propagation neural network (BPNN). In this study, statistical features were extracted from bioelectrical impedance and PPG signals, and a BPNN, which has the advantages of weight modification and wide application range, was used as the blood glucose prediction model. The remainder of this paper is structured as follows. Section 2 describes the physiological parameter feature extraction process. Section 3 describes analyses of measurement data preprocessing technology used in this study. Section 4 presents the experimental method and system configuration. Section 5 gives analyses and discussion of network experiment results. Finally, Section 6 presents the conclusions of this study.

## 2. Physiological Parameter Feature Extraction

When red and near-infrared light are transmitted into human tissue, the glucose contained in one’s blood absorbs the lights at exactly this wavelength, allowing the sensor to transforms the absorbed light into an electrical current and, subsequently, into voltage readings. Such voltage readings are referred to as the PPG signal. Protein in the human body naturally contains net charge and dipoles. A dipole is formed by positive and negative charges, while the net charge represents the excessive portion of positive or negative charges. An increase in glucose concentration would impact the structure of the protein, and thereby trigger a change in dipole, which can be captured by the bioelectrical impedance sensor.

In this study, the MAX86150 Evaluation System (Maxim Integrated, San Jose, CA, USA) was used to measure PPG signals. The current adjustment range of the hardware was 0–100 mA, and the current pulse width was 50–400 µs. It included two light sources, one of red light at 670 nm and one of infrared light at 900 nm, enabling the simultaneous measurement of different wavelengths of PPG signals. To obtain the bioelectrical impedance values, an EVAL-AD5933EBZ Evaluation Board (Analog Devices, Wilmington, MA, USA) was used. The board had a measurement frequency of 1–100 kHz, and the measurement frequency range could be easily adjusted. The maximum sampling times reached 512 times to yield bioelectrical impedance values at different frequencies.

To determine the stability and robustness of the proposed system, the blood glucose values measured by the commercially available noninvasive glucose monitor ESER GlucoGenius (HK Eser Int’l Tech Development Co., Ltd., Hong Kong, China) were used as the target network of network training. According to the Clarke EGA, the verification similarity reached 87.56%. In the experiment, the dual PPG signals, bioelectrical impedance values, and blood glucose values were measured, and the signal processing method used in [18] was referenced. The peaks of the PPG signals were used to calculate the statistical characteristics of mean, variance, skewness, kurtosis, standard deviation, and message entropy.

These characteristics were defined as follows: The mean is a measurement value often used in statistics. In this study, we acquired the values of the peaks of the PPG signals and divided them by the number of peaks to obtain the mean value of the peaks. Variance is the average distance of all values from the mean. In this study, the peaks were used to calculate the variance. Skewness is the distribution of the samples used for judgment. In this study, kurtosis was used to measure the peak value of the probability distribution of the random variables of the data. Standard deviation is used to calculate the degree of dispersion of a data set, with a high standard deviation indicating a large gap between most values and the mean, a low standard deviation indicating that most values exhibited little difference from the mean. Information entropy is expressed as the expected values contained in all categories.

In [17], the measurement frequency of bioelectrical impedance ranged from 50 to 100 kHz, with an interval of 5 kHz. The present study used bioelectrical impedance data measured by currents of 11 frequencies, with the bioelectrical impedance data of each frequency involving a real part, imaginary part, phase, and amplitude values. This study referenced [17,19,20,21,22] and used the patient characteristics of age, height, weight, heart rate, blood flow velocity, hemoglobin, and blood oxygen saturation as the physiological parameters for blood glucose estimation.

## 3. Analyses of Measurement Data Preprocessing Technology

### 3.1. Motion Artifact Suppression Technology

**Figure 1:**
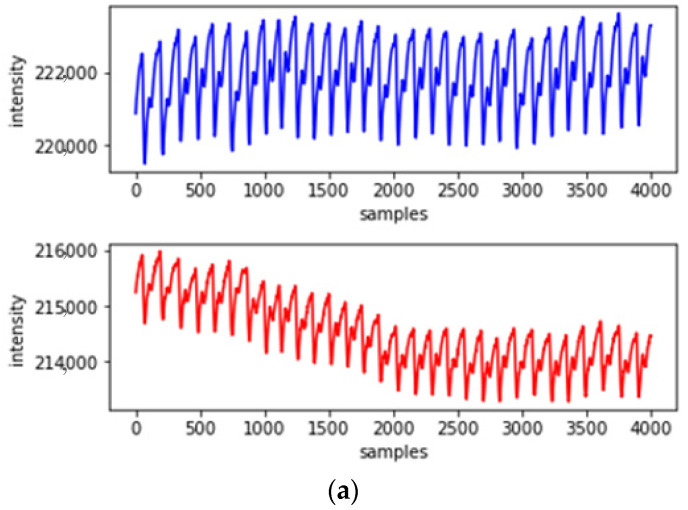

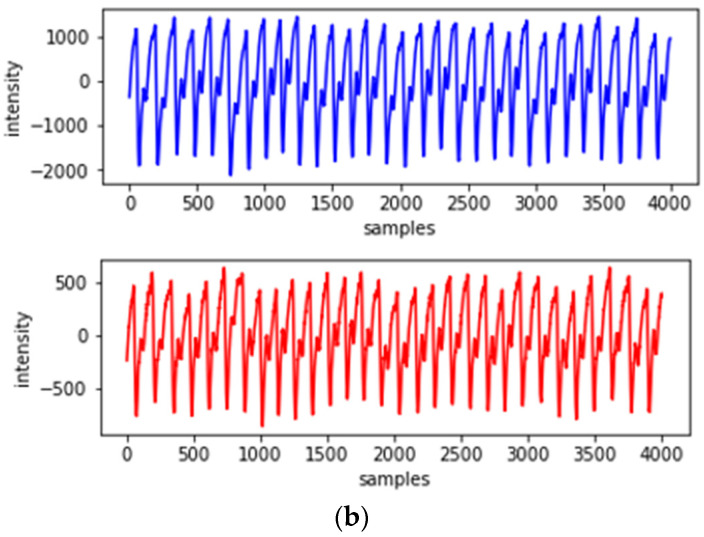
Dual-wavelength photoplethysmography (PPG) measurement waveform (blue line denotes red light, red line denotes infrared light); (**a**) original unprocessed PPG waveform; (**b**) PPG waveform filtered by a Savitzky–Golay filter.

Interference by motion artifacts causes changes in the waveform shape of PPG signals, which lead to corresponding changes in their statistical data. Therefore, we used SG filtering to remove the offset value caused by motion artifacts and subtracted the offset value from the measured PPG signal, thereby acquiring a PPG signal with reduced motion artifact effects [23]. SG filtering is an efficient smoothing and denoising method that performs a weighted moving average, with the weighting implemented as a higher level of polynomial. One of the biggest advantages of this filter is that it preserves the salient features of the maxima and minima that would normally be corrupted by other smoothing filters. Figure 1a illustrates the PPG measurement waveforms at different wavelengths that are affected by motion artifacts; Figure 1b displays PPG signals of different wavelengths with the use of an SG filter that filters the motion artifacts.

### 3.2. Use of Principal Component Analyses to Reduce Dimensionality

To achieve a more efficient model training, principal component analyses were used to reduce the dimensionality of the bioelectrical impedance dataset and thereby minimize information loss and substantially decrease the amount of computation required [24]. The processing method consisted of five steps.

(1) Normalize and subtract the mean value from the data;
(2) Calculate the covariance matrix through eigenvalue decomposition;
(3) Calculate the eigenvalues and eigenvectors of the covariate matrix;
(4) Select features and establish eigenvectors;
(5) Map the original data to the selected principal component space to obtain the data after dimensionality reduction.

This reduced the amount of input data for computation and left useful measurement signal characteristics for network use.

## 4. Experimental Method and System Configuration

### 4.1. Research Participants

This study was a collaboration between the authors of this paper and physicians from National Taiwan University Hospital Yunlin Branch, Taiwan, and was approved for a clinical trial by the Institutional Review Board. A total of 40 participants consisting of volunteers from National Taiwan Ocean University were recruited. The inclusion criteria were students with normal blood pressure and aged 18 years or older. Pregnant women, minors (under the age of 18), and individuals with mental disabilities were excluded.

Table 1 presents the participants’ age, height, heart rate, blood flow velocity, hemoglobin, blood oxygen saturation, and blood glucose range.

**Figure 2:**
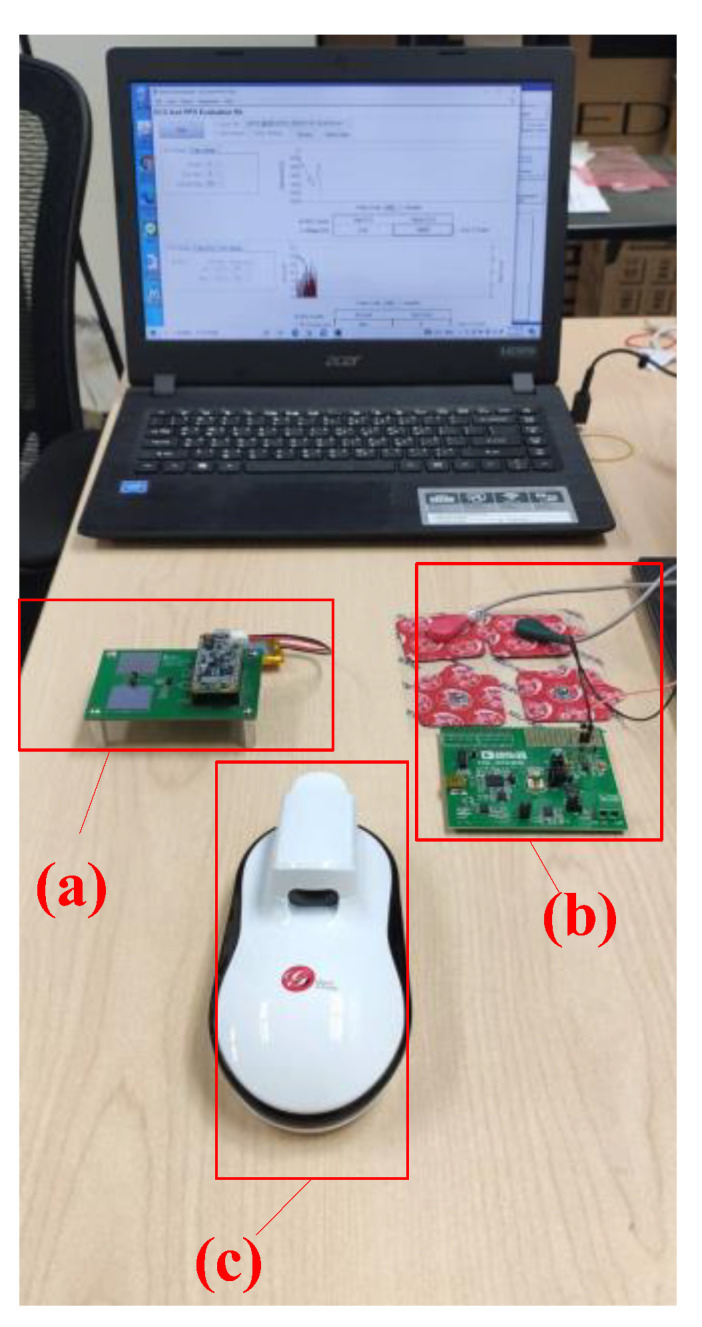
Experimental environment and equipment layout: (**a**) the PPG waveform measuring instrument; (**b**) the bioelectrical impedance value measuring instrument; (**c**) a commercially available noninvasive glucose meter.

The experiment location was the Smart IoT Laboratory of the Department of Electrical Engineering, National Taiwan Ocean University. The experimental environment and measuring instruments are presented in Figure 2. Before the experiment began, the detailed process and experimental purpose were explained to the patients, and the experiment commenced after the patients signed a consent form. During data collection, patients were asked to sit in a comfortable chair and not talk to or make physical contact with anyone.

**Figure 3:**
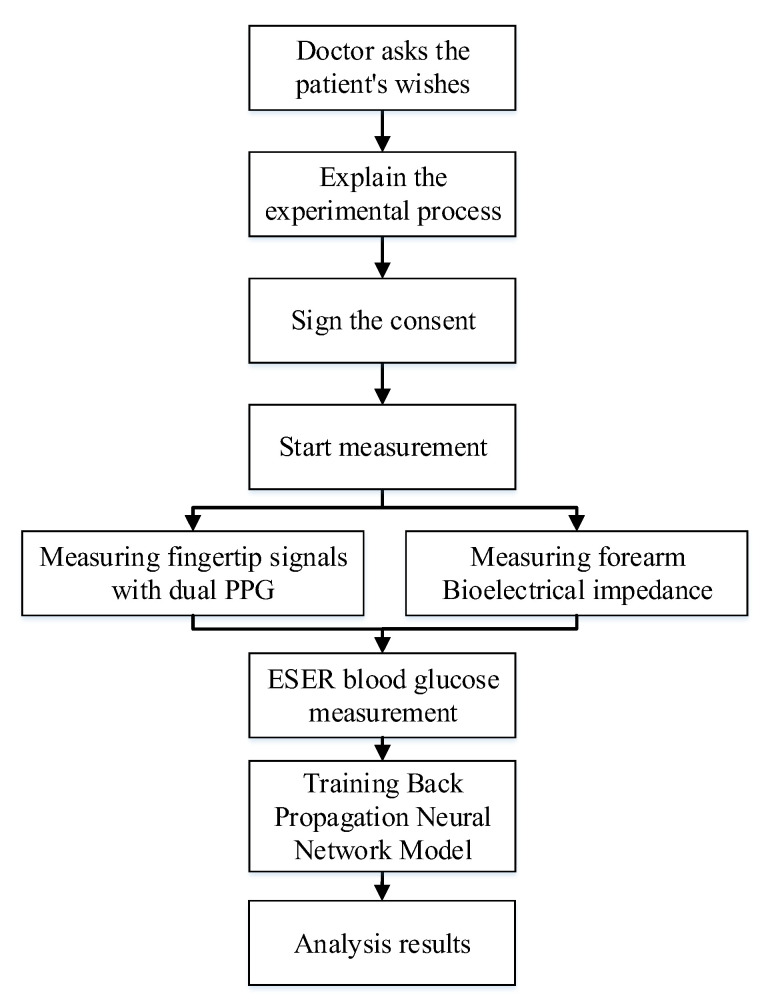
Flow chart of the clinical trial.

This study tested the noninvasive measurement methods developed, namely dual-wavelength PPG measurement and bioelectrical impedance measurement, to obtain blood glucose-related characteristics. The experimental flow chart is presented in Figure 3. The participants’ data were collected using the following methods:

(1) Explain the research protocol and experimental method to each volunteer, and confirm that they met the inclusion criteria, after which they are asked to sign a consent form.
(2) Participants placed their hands flat and at the same height as their hearts and sat still quietly for 3 min while PPG waveforms and bioelectrical impedance values were being collected.
(3) After the PPG waveform and bioelectrical impedance measurement, the commercially available noninvasive glucose meter ESER GlucoGenius was used to conduct a 2 min measurement to obtain participants’ blood glucose values.
(4) After the experiment, the participants’ characteristics such as age, height, weight, heart rate, blood flow velocity, hemoglobin, and blood oxygen saturation were obtained for neural network use.

### 4.2. Experimental System Architecture Diagram

**Figure 4:**
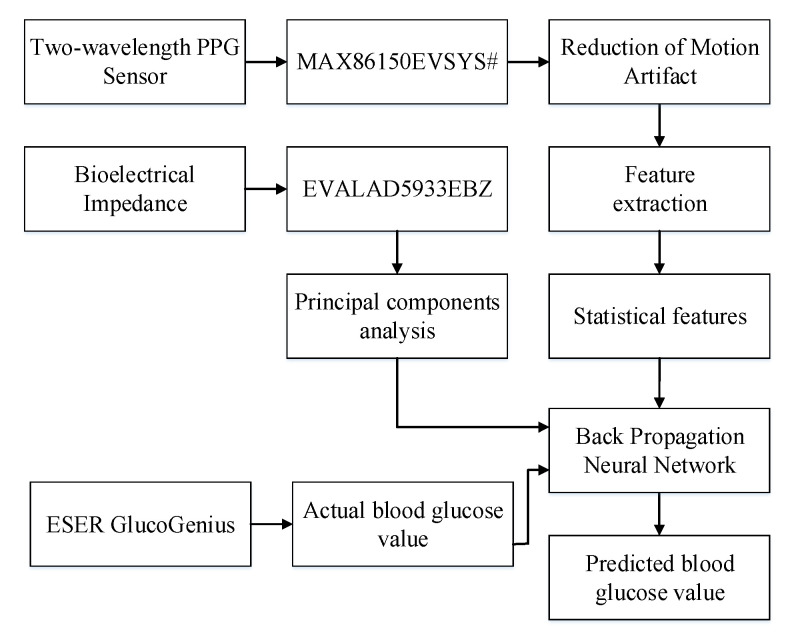
Experimental system architecture.

The experimental system architecture presented in Figure 4 included a MAX86150 Evaluation System that uses 660 nm red light and 900 nm infrared light, an EVAL-AD5933EBZ Evaluation Board bioelectrical impedance measuring device, and an ESER GlucoGenius noninvasive glucose meter. This system obtained PPG signals by having the participants place their left middle fingers on the sensor and attaching two electrode patches (10 cm apart) under their left wrists, simultaneously obtaining the dual-wavelength PPG signals and the bioelectrical impedance values. After the measurement was complete, the ESER GlucoGenius noninvasive glucose meter was used to measure the participants’ blood glucose levels using their right hands. The measured data were analyzed and calculated using an algorithm, after which the mean, variance, skewness, kurtosis, standard deviation, and information entropy were obtained, which were used by the BPNN to calculate the blood glucose level.

### 4.3. Back-Propagation Neural Network Design

**Figure 5:**
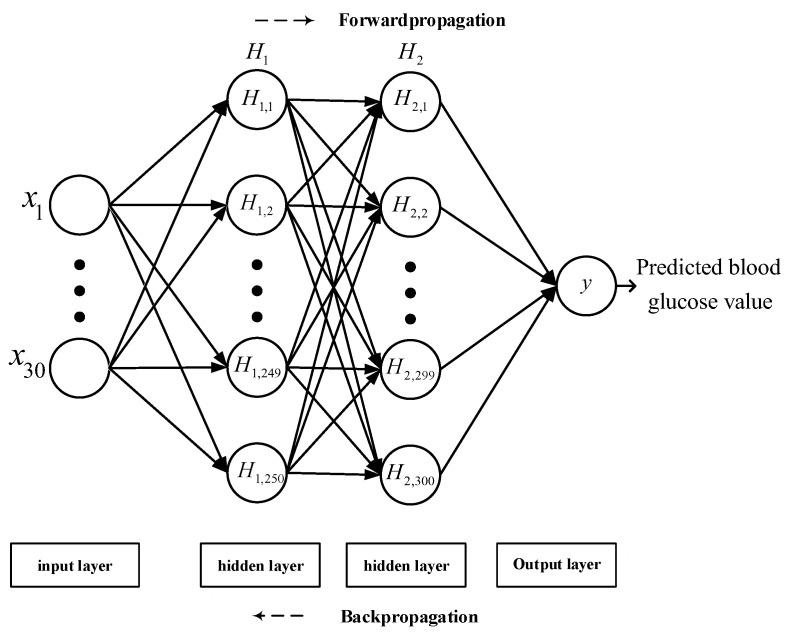
Proposed BPNN architecture.

The architecture of the BPNN is illustrated in Figure 5. Two PPG signals of different wavelengths were used to calculate six types of statistical features. The characteristic quantities of the six types of statistical features and bioelectrical impedance were 12 × 40 and 11 × 40, respectively, and the number of volunteer physiological features were 7 × 40. The statistical features of the PPG data and bioelectrical impedance features were concatenated to acquire 30 × 40 values, which were used as the input of the BPNN.

The features described in Table 2 are all input features of the BPNN. The input features were first weighted and adjusted by 250 neurons through the hidden layer of the first layer and were then weighted by 300 neurons in the second hidden layer. Because the output data only contained blood glucose values, to avoid the network failing to converge or overfitting, the number of iterations and learning rate were considered when designing the network. Because an excessively low or high number of iterations would lead to incomplete network training and overfitting, respectively, repeated tests revealed that setting the number of iterations to 550 could produce more favorable network performance. The learning rate was set to 0.01 in the beginning, producing a faster convergence for the initial network training and avoiding entering a locally optimal solution in the beginning. Furthermore, the learning rate was set to decrease after every ten iterations, facilitating a stable convergence when the BPNN performed the gradient descent due to errors and producing a higher probability of obtaining the global optimal solution.

### 4.4. Model Performance Evaluation

To evaluate the performance of the BPNN, we used the following quantitative evaluation metrics:

(1) Mean squared error (MSE): The regression loss function used in machine learning, also known as L2 loss. It can judge the degree of change in the data through the sum of squares of the distance between the actual value and the predicted value. Therefore, a smaller MSE indicates a more favorable accuracy of model prediction.
(2) Root mean squared error (RMSE): RMSE is used to measure the deviation between the actual quality and the predicted value. By calculating the sum of squares of the distance between the actual value and the predicted value, RMSE is equivalent to the square root of MSE, and its effect is to produce a more favorable description of the data.
(3) Mean absolute error (MAE), also known as L1 loss: After taking the absolute value of all actual values and predicted values, the arithmetic mean is obtained. The presence of positive and negative errors during error calculation provides opportunities for the two types of errors to offset each other. Therefore, the absolute value is added for evaluation.
(4) Mean absolute relative difference (MARD): MARD is an indicator used to assess continuous blood glucose monitoring. A lower value of the average difference between the actual and predicted values signifies a higher accuracy of the designed instrument.
(5) Coefficient of determination (*R*^2^): The coefficient of determination is used to indicate the similarity of the actual data to the predicted data. A value between 0 and 1 is obtained by dividing the predicted variable by the target variable, with a value closer to 1 representing higher similarity.
(6) Clarke EGA: Clarke EGA is the standard to determine the accuracy of blood glucose meters, which is achieved by quantifying the blood glucose values obtained by the glucose meter and comparing them with reference values. The grid consists of five regions. The values in region A indicate that the blood glucose level can be determined and used to enable the patient to receive the appropriate treatment. Region B indicates that the values have a large deviation from the reference values but will not cause adverse effects if they are used to determine treatments. Regions C, D, and E indicate that the values have deviated to the extent that should treatment be based on the values, the treatment will be unnecessary or harmful. Therefore, general precision blood glucose meter measurements should fall in regions A or B of the Clarke EGA.

The aforementioned performance evaluation indicators were adopted to evaluate whether the proposed blood glucose calculation system could obtain accurate measurements.

## 5. Analyses and Discussion of Network Experiment Results

**Figure 6:**
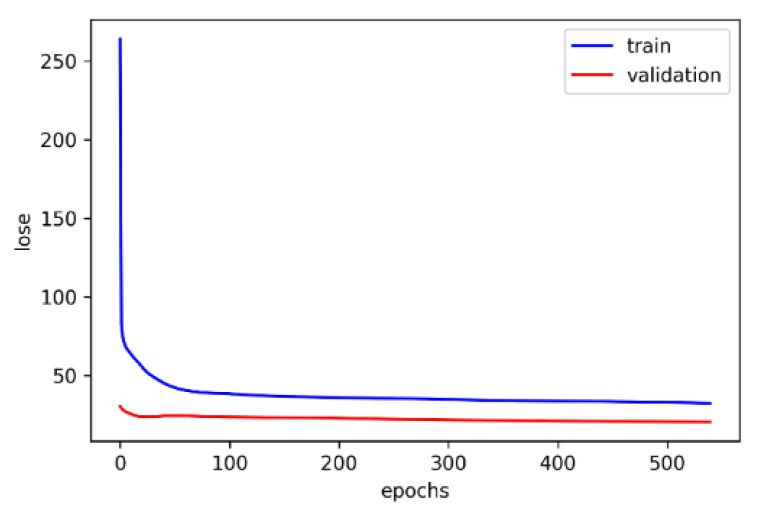
Training convergence curve.

A total of 40 participants were recruited in the experiment, and 3 min of data were collected from each participant. PPG signals were measured continuously, and bioelectrical impedance was measured once per minute. Twelve statistical features were calculated from the measured PPG signals. The measured bioelectrical impedance data were from 11 frequency bands, and each frequency band had four features; principal component analyses were then conducted to reduce their dimensionality to 11 features. The participants’ seven physiological state features were added to produce a total of 30 features, which were used as the network rate training and verification. Figure 6 illustrates the training and validation curves of the BPNN. The training loss curve was observed to decrease rapidly before reaching 50 iterations, and the subsequent curve was more stable and exhibited less fluctuation. Accordingly, the proposed BPNN model possessed stability and robustness, and could stably converge data collected by the dual-wavelength PPG sensor and bioelectrical impedance measurement. Furthermore, the early stopping method avoided over-training of the model. In comparison with [12,13,15,16,17,21,25,26,27], which served as the standard for predicting blood glucose, the proposed blood glucose estimation system had an MSE, RMSE, MAE, MARD, and $R^{2}$ of 40.736, 6.3824, 5.0896, 4.4321, and 0.997, respectively, all of which fell within region A of the Clarke EGA.

**Figure 7:**
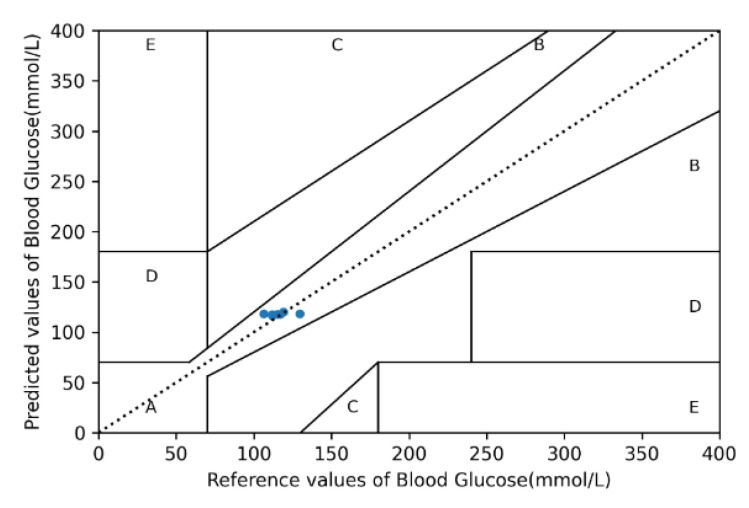
Clarke error grid.

Figure 7 illustrates the Clarke grid analyses plotted using the network verification of 30 features obtained from the dual-wavelength PPG measurement data, bioelectrical impedance from 11 frequencies, and physiological features. The points predicted in the figure were all located in region A, indicating that the model designed by this system had an extremely high correlation with existing methods in blood glucose assessment.

A comparison with the results of systems developed in other studies is presented in Table 3. The results reveal that the performance obtained by the proposed method was superior to those of the other systems, verifying the effectiveness of the proposed architecture.

## 6. Conclusions

The proposed blood glucose detection method in this study involved the measurement of dual-wavelength PPG and bioelectrical impedance. The dual-wavelength PPG sensor was placed between the index and middle fingers of the participants’ left hands, and two electrode patches were placed 10 cm apart under the participants’ left wrists to achieve the goal of simultaneously capturing signals. The two patches separately recorded the complete continuous waveforms of the two PPGs, which were then converted into statistical features such as mean, variance, skewness, kurtosis, standard deviation, and message entropy, totaling 12 features. In terms of the bioelectrical impedance, data in the frequency range of 50 and 100 kHz were recorded. Specifically, data on the real part, imaginary part, phase, and amplitude of 11 frequencies were acquired by sampling every 5 kHz, resulting in a total of 44 features. To enhance the robustness of the network model, SG filtering was used in PPG processing during data preprocessing to improve baseline drift caused by motion artifacts. For bioelectrical impedance processing, dimensionality reduction was achieved by using PCA to retain crucial information and reduce the computational complexity. Finally, the blood glucose values were calculated using the proposed BPNN algorithm.

The input network training database used in this paper had a total of 40 participants. The evaluation indicators MSE, RMSE, MAE, MARD, and $R^{2}$ of the blood glucose values predicted by the proposed model were 40.736, 6.3824, 5.0896, 4.4321, and 0.997, respectively, all of which fell within region A of the Clarke EGA. Regardless of whether the method proposed in this study used solely PPG, solely bioelectrical impedance, or the combination of both PPG and bioelectrical impedance, the results were more favorable compared with the results obtained in other studies, verifying the measurement accuracy of the proposed system. The selection and design of deep learning networks is one of the future work items. While the group method of data handling (GMDH) neural network and our proposed network are identical in terms of network structure, the GMDH neural network is equipped with three additional functions, such as automatic check on neuron, hidden layer counts, and automatic search of effective feature input. In particular, the function of automatic search of effective feature input would be especially useful when training networks with unidentified features, given the importance of effective feature value under the setting of online training. These kinds of networks are one of the options that can be used in our research.

## Figures and Tables

**Table 1:** Physiological characteristics of the participants.

Parameters	Daily Activity
Age Range (years)	18–25
Height (cm)	165 ± 20
Weight (kg)	65 ± 30
Heart rate (bpm)	70 ± 28
Blood flow rate (mm/s)	280 ± 100
Hemoglobin (g/L)	160 ± 20
Pulse oximetry (%)	94 ± 5
blood glucose (mg/dL)	110 ± 15

**Table 2:** Input characteristic values used by the BPNN.

1	Infrared light PPG mean	2	Red light PPG mean	3	Infrared light variance
4	Infrared light PPG variance	5	Infrared light PPG skewness	6	Red light PPG skewness
7	Infrared light PPG kurtosis	8	Red light PPG kurtosis	9	Infrared light PPG standard deviation
10	Red light PPG standard deviation	11	Infrared light PPG Information Entropy	12	Red light PPG Information Entropy
13	Age (years)	14	Height (cm)	15	Weight (kg)
16	Heart rate (bpm)	17	Blood flow rate (mm/s)	18	Hemoglobin (g/L)
19	Pulse oximetry (%)	20	Frequency 50k Bioelectrical Impedance values	21	Frequency 55k Bioelectrical Impedance values
22	Frequency 60k Bioelectrical Impedance values	23	Frequency 65k Bioelectrical Impedance values	24	Frequency 70k Bioelectrical Impedance values
25	Frequency 75k Bioelectrical Impedance values	26	Frequency 80k Bioelectrical Impedance values	27	Frequency 85k Bioelectrical Impedance values
28	Frequency 90k Bioelectrical Impedance values	29	Frequency 95k Bioelectrical Impedance values	30	Frequency 100k Bioelectrical Impedance values

**Table 3:** Comparison of noninvasive blood glucose assessment systems.

Reference	Modality	MSE	RMSE	MAE	MARD	$R^{2}$	Clarke EGA
Hina et al. [12]	NIRS	N/A	11.20	N/A	7.62%	0.937	95% in the A area
Hina et al. [25]	NIRS	N/A	10.20	N/A	6.9%	0.955	N/A
Gupta et al. [21]	NIRS	N/A	N/A	N/A	N/A	0.88	N/A
Guzman et al. [26]	NIRS	N/A	18.6621	16.4540	N/A	N/A	N/A
Zhu et al. [27]	NIRS	N/A	N/A	N/A	5.453%	0.936	98.413% in the A area
Zeng et al. [13]	BIS	N/A	N/A	N/A	N/A	0.99	N/A
Nanayakkara et al. [16]	BIS + NIRS	N/A	10.24	N/A	N/A	0.58	90% in the A area
Pathirage et al. [17]	BIS + NIRS	N/A	N/A	N/A	9.3%	N/A	86.1% in the A area
Fouad et al. [15]	BIS + NIRS	N/A	N/A	N/A	N/A	0.918	100% in the A area
This work	BIS + NIRS	40.736	6.3824	5.0896	4.4321%	0.9970	100% in the A area

## Data Availability

Not applicable.
